# Depressive symptoms and antidepressant use in relation to white blood cell count among postmenopausal women from the Women’s Health Initiative

**DOI:** 10.1038/s41398-024-02872-5

**Published:** 2024-03-21

**Authors:** Hind A. Beydoun, May A. Beydoun, Sylvia Wassertheil-Smoller, Nazmus Saquib, JoAnn E. Manson, Linda Snetselaar, Jordan Weiss, Alan B. Zonderman, Robert Brunner

**Affiliations:** 1 Department of Research Programs, Fort Belvoir Community Hospital, Fort Belvoir, VA USA; 2 Laboratory of Epidemiology and Population Sciences, National Institute on Aging, NIA/NIH/IRP, Baltimore, MD USA; 3 Department of Epidemiology and Population Health, Albert Einstein College of Medicine, Bronx, NY USA; 4 College of Medicine, Sulaiman AlRajhi University, Al Bukairiyah, Kingdom of Saudi Arabia; 5 Division of Preventive Medicine, Brigham and Women’s Hospital, Harvard Medical School, Boston, MA USA; 6 Department of Epidemiology, College of Public Health, University of Iowa, Iowa City, IA USA; 7 Department of Demography, UC Berkeley, Berkeley, CA USA; 8 Department of Family and Community Medicine (Emeritus), School of Medicine, University of Nevada (Reno), Reno, NV USA

**Keywords:** Depression, Physiology

## Abstract

Inflammation can play a role in the pathophysiology of depression, and specific types of antidepressants may have inflammatory or anti-inflammatory properties. Furthermore, depression and antidepressant use has been linked to white blood cell (WBC) count, a routinely measured inflammatory marker. We examined the cross-sectional and longitudinal relationships of depressive symptoms and/or antidepressant use with WBC count among postmenopausal women. Analyses of cross-sectional data at enrollment were performed on 125,307 participants, 50–79 years of age, from the Women’s Health Initiative Clinical Trials and Observational Studies who met eligibility criteria, and a subset of those with 3-year follow-up data were examined for longitudinal relationships. Depressive symptoms were defined using the Burnam Algorithm whereas antidepressant use was defined using therapeutic class codes. WBC count (Kcell/ml) was obtained through laboratory evaluations of fasting blood samples. Multivariable regression modeling was performed taking sociodemographic, lifestyle and health characteristics into consideration. At enrollment, nearly 85% were non-users of antidepressants with no depressive symptoms, 5% were antidepressant users with no depressive symptoms, 9% were non-users of antidepressants with depressive symptoms, and 2% were users of antidepressants with depressive symptoms. In fully-adjusted models, cross-sectional relationships were observed whereby women in the 2^nd^ (OR = 1.06, 95% CI: 1.01, 1.13), 3^rd^ (OR = 1.06, 95% CI: 1.00, 1.12) or 4^th^ (OR = 1.10, 95% CI: 1.05, 1.17) quartiles of WBC count were more likely to exhibit depressive symptoms, and women in the 4^th^ quartile were more likely to be users of antidepressants (OR = 1.07, 95% CI: 1.00, 1.15), compared to women in the 1^st^ quartile. Compared to women who exhibited no depressive symptoms at either visit, those with consistent depressive symptoms at enrollment and at 3-year follow-up had faster decline in WBC count (β = −0.73, 95% CI: −1.33, −0.14) over time. No significant bidirectional relationships were observed between changes in depressive symptoms score and WBC count over time. In conclusion, depressive symptoms and/or antidepressant use were cross-sectionally related to higher WBC counts among postmenopausal women. Further evaluation of observed relationships is needed in the context of prospective cohort studies involving older adult men and women, with repeated measures of depression, antidepressant use, and WBC count.

## Introduction

Depression is a frequently under-recognized [1–4] mental and behavioral issue of public health significance among women and older adults. Sex differences have been observed whereby women tend to have higher rates of internalizing disorders (i.e., depression, anxiety), while men experience more externalizing symptoms (i.e., violence, substance abuse), thereby explaining the higher prevalence of depression among women as compared to men [5–7]. In a recent meta-analysis of 48 studies, the prevalence of depression among older adults was estimated to be 28.4% [8]. Personal and situational attributes that have been linked to depression in older populations include female sex, increasing age, being single or divorced, lower education, unemployment, low income, lack of health insurance, smoking, chronic conditions and poor health, among others [3]. Previous studies have also established depression as a risk factor for a range of age-related chronic conditions including metabolic syndrome, diabetes mellitus, cardiovascular disease (CVD), and cancers [9].

Different classes of antidepressants (e.g. Selective Serotonin Reuptake Inhibitors [SSRIs], Tricyclic Antidepressants [TCAs]) are primarily prescribed to treat depression, but may be prescribed for other indications [10]. The long-term effects of antidepressants have not been adequately studied despite the fact that a quarter of those prescribed these medications take them for ≥10 years [11]. Although antidepressants can be effective at reducing depressive symptoms, especially in major depressive disorder (MDD), and potentially improving cognitive function and quality of life, they have been linked to side-effects such as weight gain, hyponatremia, reduced bone mineral density, tremor, sexual dysfunction, lessened general well-being, suicide, as well as increased risks of falls, fractures, and cardiovascular morbidities [4, 12]. Furthermore, meta-analyses of U.S. Food and Drug Administration trials suggested that antidepressants were only marginally efficacious compared to placebo despite publication bias potentially inflating their apparent efficacy [13, 14]. For the aforenoted reasons, it is important to examine the separate and joint contributions of depression and antidepressant use to age-related chronic conditions and their underlying processes.

Diet [9, 15–19], inflammatory responses [14, 20–22], neurotoxicity [18, 19], and epigenetic changes [23] are among the mechanisms that may mediate associations of depression and/or antidepressants with health problems. Depression may be associated with neuro-endocrine and neuro-inflammatory mediators as well as lifestyle, social engagement, and adherence to medical recommendations [16]. Whereas inflammation can play a role in the pathophysiology of depression and chronic conditions, specific antidepressants may have anti-inflammatory properties [10].

Postmenopausal women are at high risk for both depression and for antidepressant use, as depression increases with age and predominantly affect females [3]. Although sex differences in relationships between depression and inflammatory markers have been previously reported [24–30], little is known about the unique nature of these relationships after menopause. The Women’s Health Initiative (WHI) studies enrolled a cohort of >160,000 postmenopausal women with long-term follow-up allowing for the evaluation of cross-sectional and longitudinal relationships between depression, antidepressant use, and inflammatory markers. Evidence from the WHI suggests that depression or antidepressant use may be associated with vulnerability to age-related health problems including weight gain [17, 31], diabetes mellitus [32, 33], pre-hypertension and hypertension [34], cardiovascular disease [17, 35, 36], cognitive dysfunction [12, 37], colorectal cancer [10], bone loss and fracture [38], hip and knee osteoarthritis [39], Parkinson’s disease [40], as well as frailty [4], with detrimental impact on cancer survivorship [16, 41], all-cause and cause-specific mortality [16, 35, 41] risks. Several WHI studies have examined inflammatory markers in relation to depression, antidepressant use and chronic conditions among postmenopausal women [17, 42–46]. These inflammatory markers often consisted of the C-reactive protein (CRP), cytokines and genetic markers of inflammation which are not the most frequent or common tests in clinical settings [17, 42–45, 47, 48]. By contrast, white blood cell (WBC) [leukocyte] count is less often used as an inflammatory marker but it is a routine clinical test and was measured on the majority of WHI participants at baseline [43, 44, 49, 50]. Evidence from WHI studies suggests that inflammatory markers – particularly WBC count – may or may not be important beyond traditional risk factors as predictors of cardiometabolic conditions, including diabetes mellitus, cardiovascular disease, and stroke [43, 44, 49, 50]. More limited evidence also suggests that WBC count may be linked to depression and/or antidepressant use [51–54]. To our knowledge, WHI studies have not specifically examined depression and antidepressant use in relation to the WBC count as an inflammatory marker. We performed analyses of existing data from the Women’s Health Initiative Clinical Trials and Observational Studies (WHI-CTs and WHI-OS) to examine the cross-sectional and longitudinal associations of depression and/or antidepressant use with WBC count among postmenopausal women.

## Materials and methods

### Data source

The WHI is a long-term study focused on strategies for preventing heart disease, breast, and colorectal cancers as well as osteoporosis in postmenopausal women. The WHI study design, eligibility criteria, recruitment methods and measurement protocols are described elsewhere [55, 56]. Briefly, the WHI collected data on a multiethnic sample of postmenopausal women who were recruited and enrolled between 1993 and 1998 at 40 geographically diverse clinical centers (24 states and the District of Columbia) in the United States. The WHI study received institutional review board approval with informed consent from all participating clinical centers. WHI-CTs (*n* = 68,132) and WHI-OS (*n* = 93,676) are two components of the WHI (*n* = 161,808). Whereas WHI-CTs evaluated outcomes of menopausal hormone therapy (Hormone Therapy [HT] Trials), calcium and vitamin D supplementation ([CaD] Trial), and a low-fat eating pattern (Dietary Modification [DM] Trial), the WHI-OS evaluated causes of morbidity and mortality in postmenopausal women. At enrollment (1993–1998), WHI participants, 50–79 years of age, completed the same self-administered questionnaire covering demographics, general health, clinical and anthropometric characteristics, functional status, healthcare behaviors, reproductive, medical, and family history, personal habits, thoughts and feelings, therapeutic class of medication, hormones, supplements, and dietary intake, and many of these components were assessed again at specified follow-up timepoints.

### Design and participants

Data analyses of WHI-CT and WHI-OS participants, 50–79 years of age at enrollment (1993–1998), were performed using available data on pertinent variables described below. Accordingly, WHI participants were excluded from the analysis if they had missing enrollment data on: [1] the 6-item Center for Epidemiologic Studies Depression Scale (CES-D) and/or the 2-item Diagnostic Interview Schedule (DIS) used to define depressive symptoms according to the Burnam Algorithm; [2] Antidepressant use based on self-reported medications data; [3] WBC count based on tests performed at local clinical center laboratories using fasting blood samples; [4] sociodemographic, lifestyle and health characteristics used as covariates in multivariable analyses. To define the final sample used for cross-sectional analyses, we excluded participants who satisfied any of the following conditions at enrollment: [1] History of cancer other than non-melanoma skin cancer; [2] Energy intake extremes (<500 kcal/day or >5000 kcal/day); [3] WBC count outliers. Longitudinal analyses were performed using subsamples with available data on depressive symptoms/antidepressant use and WBC count at enrollment and 3-year follow-up visits.

### Study variables

#### Depressive symptoms

A screening algorithm previously developed by Burnam et al. with scores ranging between 0 and 1 and higher scores consistent with greater burden of depressive symptoms was generated using 6 items from the 20-item CES-D scale and 2 items from the National Institute of Mental Health’s DIS. This continuous variable was dichotomized based on a pre-established threshold whereby WHI participants with a score >0.06 had strong evidence of depressive symptoms [57–61]. Studies involving the Burnam algorithm conducted in the general population, primary care and mental healthcare settings suggested adequate sensitivity and positive predictive values for detecting depressive disorder, especially for recent disorders and those that met full DSM-III criteria [62] (See Supplementary Material). Using longitudinal sub-samples, we calculated change in the continuous and dichotomous definitions of depressive symptoms between enrollment and 3-year follow-up visits, with four groups being defined as follows: consistently not reporting depressive symptoms, depressive symptoms at enrollment only, depressive symptoms at follow-up only, and depressive symptoms at enrollment and follow-up visits.

#### Antidepressant use

WHI participants were asked to bring prescription and non-prescription medication containers at enrollment and subsequent follow-up times. For medications used for >2 weeks, drug names and doses were entered into a medications database and assigned therapeutic class codes using the Master Drug Data Base (MDDB: Medi-Span, Indianapolis, IN; Medi-Span software: First DataBank, Inc., San Bruno, CA). Antidepressant use at enrollment and 3-year follow-up visits were defined as ‘yes’ or ‘no’ variables based on the following therapeutic class codes: α-2 receptor antagonists [Tetracyclics], monoamine oxidase [MAO] inhibitors, modified cyclics, SSRIs, serotonin-norepinephrine reuptake inhibitors, TCAs, miscellaneous antidepressants, and antidepressant combinations. Participants in longitudinal subsamples were categorized as consistent non-users, users at enrollment only, users at follow-up only, and consistent users of antidepressants.

#### Depression and/or antidepressant use

A categorical variable was defined by combining depressive symptoms and antidepressant use at enrollment as follows: [1] No depressive symptoms and no antidepressant use; [2] No depressive symptoms and antidepressant use; [3] Depressive symptoms and no antidepressant use; [4] Depressive symptoms and antidepressant use. Women with no depressive symptoms and no antidepressant use were treated as the referent category for this interaction variable. Alternatively, for some analyses, depression and/or antidepressant use were defined as a dichotomous variable, at both enrollment and follow-up visits.

#### White blood cell count

Trained phlebotomists collected fasting blood samples at enrollment and 3-year follow-up visits, which were immediately centrifuged and stored at −70 °C. Complete blood counts were performed within local laboratories of WHI participating clinics, and these included WBC or leukocyte count (Kcell/ml), which was defined as a continuous variable [49]. The normal number of WBCs in the blood is 4500–11,000 cells per microliter. After excluding 8 outliers considered as extreme values (≤1.2 Kcell/ml or ≥737 Kcell/ml) based on exploratory data analyses, we evaluated the distribution of WBC count, and accordingly no transformations were applied. Instead, WBC count at enrollment was examined in quartiles (1^st^ quartile: <4.8 Kcell/ml, 2^nd^ quartile: 4.8– < 5.7 Kcell/ml, 3^rd^ quartile: 5.7– < 6.8 Kcell/ml, 4^th^ quartile: ≥6.8 Kcell/ml).

#### Covariates

Based on similarly conducted WHI studies, covariates were selected as a priori confounders to be included in multivariable regression models based on their putative relationships with variables of interest. Covariates collected at enrollment included *WHI component* (WHI-CT, WHI-OS), *socio-demographic characteristics* (age [in years], race [American Indian/Alaska Native, Asian, Native Hawaiian/Other Pacific Islanders, Black, White, More than one race, Unknown/Not reported], ethnicity [Hispanic, non-Hispanic, Unknown/Not reported], education [less than high school, high school, some college, completed college or higher level], household income [<$20,000, $20,000–$49,999, $50,000–$99,999, ≥$100,000], marital status [Married/Partnered, Single, Divorced, Widowed]), *lifestyle characteristics* (smoking status [Never Smoker, Past Smoker, Current Smoker], alcohol consumption [Non-Drinker, Former Drinker, <1 drink/week, ≥1 drink/week], physical activity [Metabolic equivalent-hours/week]), and *health characteristics*, namely, body mass index (BMI), comorbid conditions (cardiovascular disease [Yes, No], hypertension [Yes, No], hyperlipidemia [Yes, No], diabetes [Yes, No]) and self-rated health [Excellent/Very Good/Good, Fair/Poor]). Trained staff collected anthropometric data, including weight [kg] and height [cm] at enrollment [63]. Weight was measured to the nearest 0.1 kg on a balance beam scale with the participant dressed in indoor clothing without shoes, while height was measured to the nearest 0.1 cm using a wall-mounted stadiometer. BMI was calculated as (weight (kg) ÷ (height^2^ (m^2^)) and further categorized as <25.0 kg/m^2^ [underweight/normal weight]; 25.0–29.9 kg/m^2^ [overweight]; and ≥30 kg/m^2^ [obese]. History of cardiovascular disease was defined in terms of previous coronary heart disease, angina, aortic aneurysm, carotid endarterectomy or angioplasty, atrial fibrillation, congestive heart failure, cardiac arrest, stroke, or transient ischemic attack. History of hypertension was defined as self-reported diagnosis or treatment for hypertension or evidence of high blood pressure based on systolic blood pressure (SBP) or diastolic blood pressure (DBP) measurements. History of diabetes was defined as physician-diagnosed diabetes or use of diabetes medications. History of hyperlipidemia was defined as using lipid-lowering medications or having been told of high cholesterol by a physician. As a sensitivity analysis, we also considered self-reported use of anti-inflammatory medications and dietary inflammatory index (DII) (See Supplementary Material) at enrollment in multivariable models.

### Statistical analysis

All statistical analyses were conducted using SAS version 9.4 (SAS Institute, Cary, NC). Summary statistics included mean ± standard deviation for continuous variables and frequencies with percentages for categorical variables. We examined bivariate associations using the Chi-square test, independent samples t-test, one-way Analysis of Variance (ANOVA), Pearson’s correlation coefficient or their non-parametric counterparts, as appropriate. Simple and multivariable linear and logistic regression models were constructed to estimate beta (β) coefficients or odds ratios (OR) with their 95% confidence intervals (CI). First, we examined the cross-sectional association of socio-demographic, lifestyle and health characteristics with WBC count, depressive symptoms, and antidepressant use at enrollment. The Cochrane-Armitage trend test was used to examine the relationship of dichotomous characteristics with WBC count defined in quartiles. Second, we constructed linear and logistic regression models to examine the cross-sectional relationship between depressive symptoms and/or antidepressant use and WBC count defined as a continuous variable or as quartiles, before and after adjustment for covariates. The potential for collinearity among covariates was evaluated using correlation matrices, and none of the correlations among covariates was greater than 0.30. WBC count was defined in quartiles to examine linear dose-response relationships with depressive symptoms, antidepressant use, as well as depressive symptoms and/or antidepressant use. Separate linear regression models were constructed for depressive symptoms, antidepressant use, as well as depressive symptoms and/or antidepressant use as predictors of WBC count defined as a continuous variable, before and after adjustment for covariates. Furthermore, separate logistic regression models were constructed for WBC count (defined as quartiles) as a predictor of depressive symptoms, antidepressant use, as well as depressive symptoms and/or antidepressant use, before and after adjustment for covariates. We assessed the longitudinal relationship between depressive symptoms and/or antidepressant use and WBC count by examining: [1] Change in WBC count between enrollment and 3-year follow-up visits in relation to patterns of depressive symptoms and antidepressant use between enrollment and 3-year follow-up visits in fixed-effects models; [2] Bidirectional associations of WBC count with depressive symptoms score compared between the enrollment and 3-year follow-up visits in mixed-effects models.

Sensitivity analyses were performed whereby WBC count was defined in tertiles and quintiles instead of quartiles to evaluate dose-response relationships, linear, quadratic, and cubic terms for WBC count were added to models to evaluate non-linear relationships, self-rated health was defined as a categorical variable, and a smaller number of covariates (i.e. age, BMI, self-rated health) was included in specific regression models. Complete cases on exposure and outcome variables were examined for percentage and patterns of missingness on covariates. Given the large sample size and small percentage of missing data on covariates, complete subject analyses were performed and results of these analyses were found to be comparable to those obtained after multiple imputation with five datasets and 10 iterations. Key findings were also comparable with and without WBC outliers. Two-sided statistical tests were conducted at α = 0.05, with familywise Bonferroni correction to account for multiple testing.

### Sample size calculations

Based on previously conducted WHI studies, we anticipated that application of pre-specified eligibility criteria will result in the loss of <10% of WHI participants, resulting in approximately 145,000 eligible WHI participants. Of those, 15% would have evidence of high depressive symptoms and 7% would be users of antidepressants [10, 16]. Assuming two-sided independent samples t-test, α = 0.05, β = 0.2, if the total sample size was 145,000, we are able to detect a mean difference in WBC count of 0.043 Kcell/ml between depressed and non-depressed women (0.15:1 ratio) with a baseline WBC count of 5.2 Kcell/ml and a common standard deviation of 2 Kcell/ml [49]. Assuming two-sided independent samples t-test, α = 0.05, β = 0.2, if the total sample size was 145,000, we are able to detect a mean difference in WBC count of 0.059 Kcell/ml between users and non-users of antidepressants (0.07:1 ratio) with a baseline WBC count of 5.2 Kcell/ml and a common standard deviation of 2 Kcell/ml [49].

## Results

### Study flowchart

**Fig. 1:**
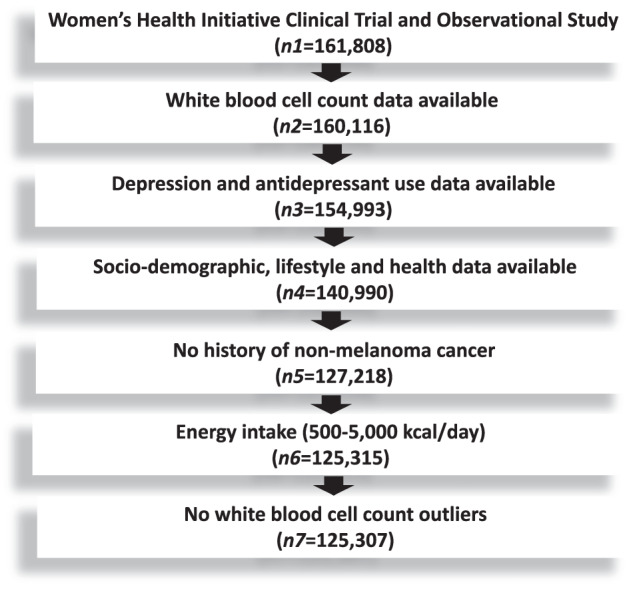
Study flowchart.

The study flowchart is displayed in Fig. 1. Of 161,808 WHI participants, 140,990 (87.1%) had non-missing data for WBC count, depressive symptoms, antidepressant use, sociodemographic, lifestyle, and health characteristics, as well as anti-inflammatory medication and DII score. Of those, 127,218 (90.2%) had no history of cancer other than non-melanoma skin cancer. After exclusion of energy intake extremes and WBC outliers, the final cross-sectional sample consisted of 125,307 (98.5%) WHI participants. Of those, 57,690 participants were included in longitudinal analyses involving depressive symptoms and WBC count and 58,370 participants were included in longitudinal analyses involving antidepressant use and WBC count.

### Descriptive statistics

Associations of WBC count, depressive symptoms, and antidepressant use with sociodemographic, lifestyle and health characteristics at enrollment are presented in Tables 1 and 2. Overall, the mean (±standard deviation) of WBC count was 6.11 (±9.96). Furthermore, WBC count varied according to most of these characteristics except for WHI component, ethnicity, educational attainment, and the DII score. Nearly 85% were non-users of antidepressants with no depressive symptoms, 5% were antidepressant users with no depressive symptoms, 9% were non-users of antidepressants with depressive symptoms, and 2% were users of antidepressants with depressive symptoms. There were significant differences in the distribution of depressive symptoms and/or antidepressant use according to all the selected characteristics.

**Table 1:** Associations of sociodemographic, lifestyle and health characteristics with white blood cell count (*n* = 125,307).

		Overall	WBC count	WBC count quartiles^a^			
		*N* (%)	Mean ± SD	1^st^	2^nd^	3^rd^	4^th^
*Total:*		125,307 (100%)	6.12 ± 9.98	24.04%	25.30%	25.34%	25.32%
*WHI component:*			*P* = 0.36	P _trend_ < 0.0001			
CT		54,058 (43.2%)	6.14 ± 9.65	40.77%	42.85%	44.11%	44.71%
OS		71,249 (56.8%)	6.09 ± 10.22	59.23%	57.15%	55.89%	55.29%
*Age (years):*			*P* = 0.0007	*P* < 0.0001			
Continuous		63.1 ± 7.2	r = 0.0096	62.25 ± 7.12	63.06 ± 7.11	63.46 ± 7.15	63.62 ± 7.19
			*P* = 0.025	*P* < 0.0001			
50–59		41,888 (33.4%)	6.02 ± 10.39	38.35%	33.53%	31.42%	30.67%
60–69		56,871 (45.4%)	6.13 ± 9.75	43.73%	45.69%	46.17%	45.87%
≥70		26,548 (21.2%)	6.23 ± 9.80	17.92%	20.79%	22.41%	23.46%
*Race:*			*P* < 0.0001	*P* < 0.0001			
American Indian/Alaska Native		374 (0.3%)	6.14 ± 1.74	0.24%	0.30%	0.32%	0.33%
Asian		3399 (2.7%)	5.54 ± 1.78	3.61%	2.85%	2.64%	1.80%
Native Hawaiian/Other Pacific Islanders		108 (0.1%)	6.09 ± 1.81	0.09%	0.07%	0.07%	0.12%
Black		10,325 (8.2%)	5.68 ± 6.12	12.04%	7.53%	6.55%	7.03%
White		107,482 (85.8%)	6.18 ± 10.59	81.58%	86.43%	87.53%	87.34%
More than one race		1479 (1.2%)	6.06 ± 2.21	1.12%	1.13%	1.18%	1.29%
Unknown/Not reported		2140 (1.7%)	6.17 ± 1.97	1.32%	1.69%	1.71%	2.09%
*Ethnicity:*			*P* = 0.32	*P* < 0.0001			
Non-Hispanic		119,147 (95.1%)	6.10 ± 10.03	95.95%	95.10%	94.76%	94.58%
Hispanic		5119 (4.1%)	6.31 ± 9.85	3.23%	4.03%	4.45%	4.59%
Unknown/Not reported		1041 (0.8%)	5.96 ± 2.44	0.82%	0.87%	0.80%	0.84%
*Marital status:*			*P* = 0.0013	*P* < 0.0001			
Married/Partnered	79,221 (63.2%)		6.03 ± 8.92	65.34%	64.87%	62.79%	59.99%
Single	5414 (4.3%)		6.29 ± 16.71	4.89%	4.24%	3.98%	4.20%
Divorced	19,687 (15.7%)		6.22 ± 10.94	15.20%	14.88%	15.81%	16.93%
Widowed	20,985 (16.8%)		6.29 ± 10.49	14.57%	16.01%	17.42%	18.88%
*Education:*			*P* = 0.12	*P* < 0.0001			
Less than high school	5949 (4.8%)		6.21 ± 7.02	4.06%	4.41%	4.87%	5.62%
High school graduate	21,625 (17.3%)		6.13 ± 7.02	15.24%	17.25%	17.64%	18.80%
Some college	47,329 (37.8%)		6.18 ± 9.66	35.29%	37.25%	38.29%	40.12%
College graduate	50,404 (40.2%)		6.03 ± 11.54	45.41%	41.08%	39.20%	35.46%
*Household income:*			*P* = 0.0016	*P* < 0.0001			
< $20,000	18,361 (14.7%)		6.35 ± 10.44	12.27%	13.29%	15.24%	17.69%
$20,000–$49,999	52,643 (42.0%)		6.13 ± 8.61	39.69%	41.37%	42.83%	44.04%
$50,000–$99,999	34,683 (27.7%)		5.97 ± 9.37	30.10%	29.01%	26.84%	24.88%
≥$100,000	11,631 (9.3%)		6.09 ± 15.27	11.63%	9.84%	8.65%	7.13%
Unknown	7989 (6.4%)		6.09 ± 15.27	6.31%	6.49%	6.45%	6.25%
*Smoking status:*			*P* < 0.0001	*P* < 0.0001			
Never	64,028 (51.1%)		5.96 ± 9.93	56.43%	53.67%	50.60%	43.95%
Past	52,748 (42.1%)		6.11 ± 10.49	41.05%	42.59%	43.16%	41.53%
Current	8531 (6.8%)		7.24 ± 6.39	2.52%	3.74%	6.24%	14.52%
*Alcohol use:*			*P* = 0.0007	*P* < 0.0001			
Non-drinker	13,370 (10.7%)		6.12 ± 10.14	10.97%	10.66%	10.51%	10.56%
Former drinker	22,850 (18.2%)		6.23 ± 7.98	16.50%	16.61%	18.24%	21.50%
<1 drink/week	41,571 (33.2%)		6.21 ± 10.17	30.38%	32.90%	33.82%	35.47%
≥1 drink/week	47,516 (37.9%)		5.97 ± 10.62	42.16%	39.83%	37.44%	32.47%
*Physical activity (Met-hours/week):*			*P* < 0.0001	*P* < 0.0001			
Continuous	12.5 ± 13.7		r = -0.019	14.39 ± 14.61	13.05 ± 13.87	12.10 ± 13.38	10.44 ± 12.59
*Body Mass Index (kg/m*^*2*^*):*			*P* < 0.0001	*P* < 0.0001			
<25	44,422 (35.5%)		5.76 ± 9.02	46.71%	37.21%	31.79%	26.66%
25–29.9	43,483 (34.7%)		6.06 ± 8.94	33.70%	36.15%	35.62%	33.28%
≥30	37,402 (29.9%)		6.59 ± 12.00	19.59%	26.64%	32.59%	40.05%
*Medical history:*							
*Cardiovascular disease:*			*P* < 0.0001	*P*_trend_ < 0.0001			
Yes	25,126 (20.0%)		6.39 ± 11.41	16.93%	18.38%	20.54%	24.19%
No	100,181 (79.9%)		6.04 ± 9.59	83.07%	81.62%	79.46%	75.81%
*Hypertension:*			*P* < 0.0001	*P*_trend_ < 0.0001			
Yes	52,859 (42.2%)		6.29 ± 8.23	33.34%	39.14%	44.29%	51.52
No	72,448 (57.8%)		5.98 ± 11.08	66.66%	60.86%	55.71%	48.48
*Diabetes:*			*P* < 0.0001	*P*_trend_ < 0.0001			
Yes	14,577 (11.6%)		6.70 ± 9.66	6.57%	9.40%	12.07%	18.24%
No	110,730 (88.4%)		6.04 ± 10.02	93.43%	90.60%	87.93%	81.76%
*Hyperlipidemia:*			*P* < 0.0001	*P*_trend_ < 0.0001			
Yes	17,186 (13.7%)		6.56 ± 13.97	10.60%	12.59%	14.70%	16.82%
No	108,121 (86.3%)		6.04 ± 9.19	89.40%	87.41%	85.30%	83.18%
*Self-rated health:*			*P* < 0.0001	*P*_trend_ < 0.0001			
Excellent/Very good/Good	114,992 (91.8%)		6.06 ± 9.84	94.18%	93.62%	91.87%	87.53%
Fair/Poor	10,315 (8.2%)		6.71 ± 11.44	5.82%	6.38%	8.13%	12.47%
*Anti-inflammatory medications:*			*P* = 0.019	*P*_trend_ < 0.0001			
Yes	22,584 (18.0%)		6.26 ± 10.43	16.06%	17.30%	18.41%	20.23%
No	102,723 (81.9%)		6.08 ± 9.88	83.94%	82.70%	81.59%	79.77%
*Dietary Inflammatory Index score:*			*P* = 0.72	*P* < 0.0001			
Continuous	1.02 ± 1.98		r = 0.00099	0.96 ± 2.00	0.98 ± 1.98	1.02 ± 1.97	1.10 ± 1.97

*SD* Standard deviation, *WBC* White Blood Cell, *WHI* Women’s Health Initiative.

^a^WBC count were defined in quartiles (1^st^ quartile: <4.8 Kcell/ml, 2^nd^ quartile: 4.8– < 5.7 Kcell/ml, 3^rd^ quartile: 5.7– < 6.8 Kcell/ml, 4^th^ quartile: ≥6.8 Kcell/ml).

**Table 2:** Associations of sociodemographic, lifestyle and health characteristics with depressive symptoms and/or antidepressant use (*n* = 125,307).

	*N* (%) or Mean ± SD				
	Overall	Depressive symptoms – No		Depressive symptoms – Yes	
		Antidepressant use		Antidepressant use	
*Total:*	125,307 (100%)	Non-user	User	Non-user	User
		105,953 (84.6%)	5996 (4.8%)	10,992 (8.8%)	2366 (1.9%)
*WHI component:*		*P* < 0.0001			
CT	54,058 (43.2%)	83.9%	5.7%	8.2%	2.2%
OS	71,249 (56.8%)	85.0%	4.1%	9.2%	1.7%
*Age (years):*		*P* < 0.0001			
Continuous	63.1 ± 7.2	63.3 ± 7.1	62.5 ± 7.1	61.7 ± 7.3	60.7 ± 7.0
		*P* < 0.0001			
50–59	41,888 (33.4%)	81.1%	5.2%	11.0%	2.7%
60–69	56,871 (45.4%)	85.7%	4.7%	7.9%	1.6%
70–79+	26,548 (21.2%)	87.6%	4.3%	6.9%	1.2%
*Race:*		*P* < 0.0001			
American Indian/Alaska Native	374 (0.3%)	73.3%	3.4%	16.6%	2.7%
Asian	3399 (2.7%)	91.9%	1.5%	6.3%	0.3%
Native Hawaiian/Other Pacific Islanders	108 (0.1%)	88.9%	0.0%	11.1%	0.0%
Black	10,325 (8.2%)	83.9%	2.6%	12.2%	1.4%
White	107,482 (85.8%)	84.6%	5.1%	8.3%	1.9%
More than one race	1479 (1.2%)	81.9%	5.1%	10.1%	2.8%
Unknown/Not reported	2140 (1.7%)	74.1%	3.9%	19.6%	2.3%
*Ethnicity:*		*P* < 0.0001			
Non-Hispanic	119,147 (95.1%)	84.9%	4.8%	8.4%	1.9%
Hispanic	5119 (4.1%)	76.6%	3.6%	17.5%	2.3%
Unknown/Not reported	1041 (0.8%)	82.8%	2.0%	13.1%	2.1%
*Marital status:*		*P* < 0.0001			
Married/Partnered	79,221 (63.2%)	86.3%	4.8%	7.3%	1.6%
Single	5414 (4.3%)	84.3%	4.9%	8.7%	2.0%
Divorced	19,687 (15.7%)	79.3%	5.5%	12.3%	2.9%
Widowed	20,985 (16.8%)	82.9%	4.2%	10.9%	1.9%
*Education:*		*P* < 0.0001			
Less than high school	5949 (4.8%)	76.6%	3.9%	16.7%	2.9%
High school graduate	21,625 (17.3%)	83.2%	4.9%	10.0%	1.8%
Some college	47,329 (37.8%)	83.7%	4.8%	9.3%	2.2%
College graduate	50,404 (40.2%)	86.8%	4.8%	6.8%	1.6%
*Household income:*		*P* < 0.0001			
<$20,000	18,361 (14.7%)	77.5%	5.0%	14.5%	2.9%
$20,000–$49,999	52,643 (42.0%)	84.6%	4.9%	8.5%	1.9%
$50,000–$99,999	34,683 (27.7%)	86.8%	4.8%	6.8%	1.6%
≥$100,000	11,631 (9.3%)	88.7%	4.5%	5.7%	1.1%
Unknown	7989 (6.4%)	84.9%	3.6%	9.9%	1.5%
*Smoking status:*		*P* < 0.0001			
Never	64,028 (51.1%)	85.9%	4.3%	8.2%	1.6%
Past	52,748 (42.1%)	84.1%	5.2%	8.7%	2.0%
Current	8531 (6.8%)	77.5%	5.4%	13.9%	3.2%
*Alcohol use:*		*P* < 0.0001			
Non-drinker	13,370 (10.7%)	85.6%	4.3%	8.5%	1.5%
Former drinker	22,850 (18.2%)	79.2%	6.5%	11.2%	3.1%
<1 drink/week	41,571 (33.2%)	84.2%	4.8%	9.1%	1.9%
≥1 drink/week	47,516 (37.9%)	87.2%	4.1%	7.4%	1.4%
*Physical activity (Met-hours/week):*		*P* < 0.0001			
Continuous	12.5 ± 13.7	12.9 ± 13.9	10.6 ± 12.8	10.2 ± 12.6	8.5 ± 11.2
*Body Mass Index (kg/m*^*2*^*):*		*P* < 0.0001			
<25	44,422 (35.5%)	87.2%	3.9%	7.6%	1.3%
25–29.9	43,483 (34.7%)	85.1%	4.8%	8.3%	1.8%
≥30	37,402 (29.9%)	80.7%	5.8%	10.7%	2.7%
*Medical history:*					
*Cardiovascular disease:*		*P* < 0.0001			
Yes	25,126 (20.0%)	80.4%	6.1%	10.8%	2.7%
No	100,181 (79.9%)	85.6%	4.5%	8.3%	1.7%
*Hypertension:*		*P* < 0.0001			
Yes	52,859 (42.2%)	83.2%	5.6%	9.1%	2.2%
No	72,448 (57.8%)	85.5%	4.2%	8.6%	1.7%
*Diabetes:*		*P* < 0.0001			
Yes	14,577 (11.6%)	79.4%	6.8%	11.0%	2.8%
No	110,730 (88.4%)	85.2%	4.5%	8.5%	1.8%
*Hyperlipidemia:*		*P* < 0.0001			
Yes	17,186 (13.7%)	81.9%	6.1%	9.5%	2.5%
No	108,121 (86.3%)	84.9%	4.6%	8.7%	1.8%
*Self-rated health:*		*P* < 0.0001			
Excellent/Very good/Good	114,992 (91.8%)	86.5%	4.4%	7.6%	1.5%
Fair/Poor	10,315 (8.2%)	62.7%	8.6%	22.2%	6.5%
*Anti-inflammatory medications:*		*P* < 0.0001			
Yes	22,584 (18.0%)	79.3%	8.2%	9.3%	3.2%
No	102,723 (81.9%)	85.7%	4.0%	8.7%	1.6%
*Dietary Inflammatory Index score:*		*P* < 0.0001			
Continuous	1.02 ± 1.98	1.00 ± 1.97	0.91 ± 1.98	1.69 ± 2.03	1.10 ± 2.04

*WHI* Women’s Health Initiative.

### Cross-sectional relationships

At enrollment, the depressive symptoms score was weakly but positively correlated with WBC count (r = 0.007, *P* = 0.007). Also, the depressive symptoms score was significantly higher among users versus non-users of antidepressants (6.44 ± 11.84 vs. 6.09 ± 9.83, *P* = 0.008). As shown in Table 3, linear regression models were constructed to evaluate the cross-sectional relationships of depressive symptoms and/or antidepressant use with WBC count. When considered separately, depressive symptoms and antidepressant use were directly related to WBC count in the unadjusted models only (*depression:* β = 0.30, 95% CI: 0.12, 0.48; *antidepressant use:* β = 0.34, 95% CI: 0.12, 0.57). When considered jointly, depressive symptoms and/or antidepressant use were directly associated with WBC count in the unadjusted (β = 0.36, 95% CI: 0.21, 0.51) and adjusted (β = 0.15, 95% CI: 0.00, 0.31) models, although the latter relationship was of borderline significance. In unadjusted models, non-users of antidepressants with depressive symptoms (β = 0.34, 95% CI: 0.15, 0.54) and users of antidepressants with no depressive symptoms (β = 0.43, 95% CI: 0.17, 0.69) experienced a higher WBC count compared to non-users of antidepressants without depressive symptoms. These relationships were statistically non-significant after adjustment for sociodemographic, lifestyle, and health characteristics. Similar results were obtained after adding use of anti-inflammatory medications and DII score to fully adjusted models (Supplementary Table S.1), and when the categorical definition of self-rated health was included in fully adjusted models (Supplementary Table S.2). As shown in Supplementary Table S.3., SSRIs was the only class of antidepressants that was associated with higher WBC count in the unadjusted model (β = 0.45, 95% CI: 0.15, 0.75), but the associations did not persist in adjusted models (β = 0.22, 95% CI: −0.04, 0.49). When depressive symptoms, antidepressant use, and depressive symptoms-by-antidepressant interaction effects were examined in a fully adjusted model, no statistically significant interaction effects were observed in relation to WBC count.

**Table 3:** Linear regression model for the cross-sectional relationship of depressive symptoms and/or antidepressant use as a predictor of white blood cell count at enrollment (*n* = 125,307).

			WBC count (Kcell/ml)	
			Unadjusted	Adjusted^a^
	*N*		β (95% CI)	β (95% CI)
*Model I:*	Yes	No		
Depressive symptoms (Yes vs. No)	13,358	111,949	0.30 (0.12, 0.48)	0.11 (−0.07, 0.29)
*Model II:*	Yes	No		
Antidepressant use (Yes vs. No)	8362	116,945	0.34 (0.12, 0.57)	0.11 (−0.11, 0.33)
*Model III:*	Yes	No		
Depressive symptoms and/or antidepressant use (Yes vs. No)	19,354	105,953		
*Model IV:*			0.36 (0.21, 0.51)	0.15 (0.00, 0.31)
Depressive symptoms /Antidepressant use (categorical)				
No depressive symptoms/No antidepressant use^b^	105,953		Ref.	Ref.
Depressive symptoms/No antidepressant use	10,992		0.34 (0.15, 0.54)	0.17 (−0.026, 0.37)
No depressive symptoms/Antidepressant use	5996		0.43 (0.17, 0.69)	0.22 (−0.04, 0.49)
Depressive symptoms/Antidepressant use	2366		0.24 (−0.16, 0.65)	−0.09 (−0.51, 0.31)

*β* Slope, *CI* Confidence Interval.

^a^Adjusted for *WHI component* (WHI-CT, WHI-OS), *socio-demographic characteristics* (age [in years], race [American Indian/Alaska Native, Asian, Native Hawaiian/Other Pacific Islanders, Black, White, More than one race, Unknown/Not reported], ethnicity [Hispanic, non-Hispanic, Unknown/Not reported], education [less than high school, high school, some college, completed college or higher level], household income [<$20,000, $20,000–$49,999, $50,000–$99,999, ≥$100,000], marital status [Married/Partnered, Single, Divorced, Widowed]), *lifestyle characteristics* (smoking status [Never Smoker, Past Smoker, Current Smoker], alcohol consumption [Non-Drinker, Former Drinker, <1 drink/week, ≥1 drink/week], physical activity [Metabolic equivalent-hours/week]), and *health characteristics*, namely, body mass index (BMI) [<25, 25– < 30, ≥30 kg/m^2^], comorbid conditions (cardiovascular disease [Yes, No], hypertension [Yes, No], hyperlipidemia [Yes, No], diabetes [Yes, No]) and self-rated health [Excellent/Very Good/Good, Fair/Poor]).

^b^Referent category.

The odds of depressive symptoms and/or antidepressant use according to quartiles of WBC count at enrollment are displayed in Table 4. In fully-adjusted models, cross-sectional relationships were observed whereby women in the 2^nd^ (OR = 1.06, 95% CI: 1.01, 1.13), 3^rd^ (OR = 1.06, 95% CI: 1.00, 1.12) or 4^th^ (OR = 1.10, 95% CI: 1.05, 1.17) quartiles were more likely to exhibit depressive symptoms, and women in the 4^th^ quartile were more likely to be users of antidepressants (OR = 1.07, 95% CI: 1.00, 1.15), compared to women in the 1^st^ quartile of WBC count. These associations remained significant after taking multiple testing into consideration. Similar results were obtained when WBC count was defined in tertiles (Supplementary Table S.4) or, in quintiles (Supplementary Table S.5). In models examining non-linear relationships, linear terms were found to be more strongly related to depressive symptoms and/or antidepressant use than quadratic and cubic terms for WBC count, supporting a linear dose-response relationship (Supplementary Table S.6).

**Table 4:** Logistic regression models for the cross-sectional relationship of white blood cell count quartiles as predictors of depressive symptoms and/or antidepressant use at enrollment (*n* = 125,307)^a^.

	*N*		Unadjusted	Adjusted^b^
			OR (95% CI)	OR (95% CI)
Model I – Depressive symptoms (Yes vs. No):	Yes	No		
*WBC count (Kcell/ml):*				
1^st^ quartile	2762	27,365	Ref.	Ref.
2^nd^ quartile	3183	28,518	1.11 (1.05, 1.17)	1.06 (1.01, 1.13)
3^rd^ quartile	3386	28,369	1.18 (1.12, 1.25)	1.06 (1.00, 1.12)
4^th^ quartile	4027	27,697	1.44 (1.37, 1.52)	1.10 (1.05, 1.17)
Model II – Antidepressant use (Yes vs. No):	Yes	No		
*WBC count (Kcell/ml):*				
1^st^ quartile	1709	28,418	Ref.	Ref.
2^nd^ quartile	1912	29,789	1.07 (0.99, 1.14)	0.97 (0.91, 1.04)
3^rd^ quartile	2119	29,636	1.19 (1.11, 1.27)	0.99 (0.93, 1.06)
4^th^ quartile	2622	29,102	1.49 (1.41, 1.59)	1.07 (1.00, 1.15)
Model III – Depressive symptoms and/or antidepressant use (Yes vs. No):	Yes	No		
*WBC count (Kcell/ml):*				
1^st^ quartile	4002	26,125	Ref.	Ref.
2^nd^ quartile	4592	27,109	1.11 (1.05, 1.16)	1.04 (0.99, 1.09)
3^rd^ quartile	4902	26,853	1.19 (1.14, 1.24)	1.04 (0.99, 1.09)
4^th^ quartile	5858	25,866	1.48 (1.42, 1.54)	1.10 (1.06, 1.16)

*CI* Confidence Interval, *OR* Odds Ratio, *WBC* White blood cells.

^a^WBC count were defined in quartiles (1^st^ quartile: <4.8 Kcell/ml, 2^nd^ quartile: 4.8– < 5.7 Kcell/ml, 3^rd^ quartile: 5.7– < 6.8 Kcell/ml, 4^th^ quartile: ≥6.8 Kcell/ml).

^b^Adjusted for *WHI component* (WHI-CT, WHI-OS), *socio-demographic characteristics* (age [in years], race [American Indian/Alaska Native, Asian, Native Hawaiian/Other Pacific Islanders, Black, White, More than one race, Unknown/Not reported], ethnicity [Hispanic, non-Hispanic, Unknown/Not reported], education [less than high school, high school, some college, completed college or higher level], household income [<$20,000, $20,000–$49,999, $50,000–$99,999, ≥$100,000], marital status [Married/Partnered, Single, Divorced, Widowed]), *lifestyle characteristics* (smoking status [Never Smoker, Past Smoker, Current Smoker], alcohol consumption [Non-Drinker, Former Drinker, <1 drink/week, ≥1 drink/week], physical activity [Metabolic equivalent-hours/week]), and *health characteristics*, namely, body mass index (BMI) [<25, 25– < 30, ≥30 kg/m^2^], comorbid conditions (cardiovascular disease [Yes, No], hypertension [Yes, No], hyperlipidemia [Yes, No], diabetes [Yes, No]) and self-rated health [Excellent/Very Good/Good, Fair/Poor]).

### Longitudinal relationships

Of 82,949 women with available data at enrollment and 3-year follow-up, 69476 were not depressed at either time point, 5239 were depressed at enrollment only, 4808 were depressed at follow-up only, and 3426 were depressed at both time points. Of 58,030 women with available data at enrollment and 3-year follow-up, 52172 were non-users of antidepressants at either time point, 2597 were users of antidepressants at enrollment only, 1345 were users of antidepressants at follow-up only, and 1916 were users of antidepressants at both time points. As shown in Table 5, there were no significant longitudinal associations between antidepressant use and change in WBC count between enrollment and 3-year follow-up visits. After adjusting for confounders, women who consistently exhibited depressive symptoms at enrollment and follow-up visits experienced a faster decline in WBC count (β = −0.73, 95% CI: −1.33, −0.14) between enrollment and follow-up visits, compared to those with no depressive symptoms at either visit. Specifically, least squares means estimate (±standard error) for change in WBC count between enrollment and 3-year follow-up visits according to patterns in depression in fully-adjusted models were as follows: consistently had no depressive symptoms (−0.32 ± 0.45, *P* = 0.48), depressive symptoms at enrollment only (−0.14 ± 0.49, *P* = 0.78), depressive symptoms at follow-up only (−0.46 ± 0.50, *P* = 0.36), and consistently had depressive symptoms (−1.05 ± 0.52, *P* = 0.045). As such, the group of women with consistently high depressive symptoms was the only one with WBC count declining between visits, after adjusting for sociodemographic, lifestyle, and health characteristics. Similar results were obtained when age, BMI and self-rated health were the only covariates included in the multivariable model (Supplementary Table S.7.). Conversely, no significant bidirectional relationships were observed between depressive symptoms score and WBC count over time. In particular, depressive symptoms scores at enrollment were not significantly related to change in WBC count over time and WBC count at enrollment were not significantly related to change in depressive symptoms scores over time (Supplementary Tables S.8 and S.9).

**Table 5:** Linear regression models for change in WBC count between enrollment and 3-year follow-up visits in relation to patterns of depressive symptoms and antidepressant use between enrollment and 3-year follow-up visits.

	WBC count (Kcell/ml)		
	β (95% CI)		
	*N*	Unadjusted	Adjusted^a^
Depressive symptoms (*n* = 56,411):			
Consistently had no depressive symptoms	47,774	Ref.	Ref.
Depressive symptoms at enrollment only	3453	0.16 (−0.30, 0.62)	0.18 (−0.29, 0.64)
Depressive symptoms at follow-up only	3069	−0.15 (−0.63, 0.33)	−0.14 (−0.64, 0.35)
Consistently had depressive symptoms	2115	−0.74 (−1.32, −0.16)	−0.73 (−1.33, −0.14)
Antidepressant use (*n* = 58,024):			
Consistent non-user (enrollment and follow-up)	52,168	Ref.	Ref.
Enrollment user only	2596	−0.26 (−0.79, 0.26)	−0.25 (−0.78, 0.28)
Follow-up user only	1344	0.13 (−0.59, 0.86)	0.17 (−0.56, 0.90)
Consistent user (enrollment and follow-up)	1916	0.12 (−0.48, 0.74)	0.16 (−0.46, 0.77)

*β* Slope, *CI* confidence intervals, *WBC* White blood cells.

^a^Adjusted for *WHI component* (WHI-CT, WHI-OS), *socio-demographic characteristics* (age [in years], race [American Indian/Alaska Native, Asian, Native Hawaiian/Other Pacific Islanders, Black, White, More than one race, Unknown/Not reported], ethnicity [Hispanic, non-Hispanic, Unknown/Not reported], education [less than high school, high school, some college, completed college or higher level], household income [< $20,000, $20,000-$49,999, $50,000-$99,999, ≥$100,000], marital status [Married/Partnered, Single, Divorced, Widowed]), *lifestyle characteristics* (smoking status [Never Smoker, Past Smoker, Current Smoker], alcohol consumption [Non-Drinker, Former Drinker, < 1 drink/week, ≥ 1 drink/week], physical activity [Metabolic equivalent-hours/week]), and *health characteristics*, namely, body mass index (BMI) [< 25, 25- < 30, ≥ 30 kg/m^2^], comorbid conditions (cardiovascular disease [Yes, No], hypertension [Yes, No], hyperlipidemia [Yes, No], diabetes [Yes, No]) and self-rated health [Excellent/Very Good/Good, Fair/Poor]).

## Discussion

In this study involving 125,307 WHI participants, 50–79 years of age, we examined cross-sectional and longitudinal relationships of depressive symptoms and antidepressant use with WBC count among postmenopausal women. Approximately 15% of women had depressive symptoms and/or were users of antidepressants at enrollment, with an average WBC count of 6.11 Kcell/ml. In cross-sectional analyses, women who exhibited depressive symptoms and/or were users of antidepressants had higher quartiles of WBC count than those who did not exhibit depressive symptoms and were non-users of antidepressants at enrollment. Women who exhibited depressive symptoms at enrollment and at 3-year follow-up visits experienced a faster decline in WBC count compared to women who did not experience depressive symptoms at either visit, implying that sustained depression may be linked to immune senescence over time. Although the recall period for the 6-item CES-D scale used in the Burnam Algorithm for depressive symptoms was the week prior to the WHI visit, we postulate that chronic exposure to cortisol might lead to leukopenia, thereby explaining why consistent depressive symptoms was found to be associated with reduced WBC count. Depressive symptoms at enrollment were not significantly related to change in WBC count over time and WBC count at enrollment was not significantly related to change in depressive symptoms scores over time. Although antidepressant users were more frequent among those in the upper versus lower quartiles of WBC count, specific classes of antidepressants were not cross-sectionally related to WBC count. Given the adjustment of numerous covariates in regression models, it is plausible that the study was underpowered to detect significant associations involving antidepressants. Also, it is worth noting that many of these antidepressants may have been prescribed for other reasons (e.g. SNRIs for postmenopausal symptoms, TCAs for migraines or neuropathy), and that examination of SSRIs may provide a more accurate understanding of factors associated with antidepressants.

The health consequences of depression and antidepressant use are of public health significance. In fact, a bi-directional relationship has been previously reported between depression and quality of life in older age groups [64]. Moreover, depression is a major contributor to the Global Burden of Diseases, Injuries, and Risk Factors (GBD) as it is the most prevalent mental disorder in older populations [3]. Prevalence, disability-adjusted life-years (DALYs), years lived with disability, and years of life lost attributed to mental disorders were evaluated, through a systematic review of multiple international databases between 1990 and 2019 [1]. The proportion of global DALYs attributed to mental disorders increased from 3.1% in 1990 to 4.9% in 2019 [1], with age-standardized DALY rates significantly higher among women than men [1]. By the same token, antidepressants are among the most widely prescribed medications among older adults [12], with antidepressant use increasing by nearly 400% between 1988–1994 and 2005–2008 [31]. Studies of the consequences of long-term antidepressant use are scant and not comprehensive, often focusing on relapse and remission rather than neurological outcomes [65, 66]. There is little consensus regarding treatment with antidepressants as opposed to alternatives such as psychotherapy or complementary medicine among individuals who do not meet the diagnostic criteria of MDD and among special populations of women and older adults [66–70].

Inflammation has been linked to many chronic conditions and strategies aimed at preventing or treating inflammation may be effective at reducing the burden of these conditions [71]. In this study, we found cross-sectional and longitudinal relationships of WBC count with depressive symptoms but only cross-sectional relationships between antidepressant use and WBC count. These findings may suggest confounding by indication whereby antidepressant use is likely a marker of disease severity [53, 72–74]. Furthermore, these findings are consistent with some of the previously conducted studies involving a wide range of populations [51–54]. For instance, Sealock et al. found a robust relationship between depression polygenic scores and WBC count by performing a meta-analysis of electronic health record data from 382,452 patients of European and African descent across 4 health care systems, with mediation analyses suggesting a bidirectional association, whereby WBC count accounted for 2.5% of the association of depression polygenic score with depression and depression accounted for 9.8% of the association of depression polygenic score with WBC count [52]. However, Mendelian randomization suggested that WBC count may increase depression risk, whereas depression did not affect WBC count [52]. Shafiee et al. examined associations of depressive and anxiety symptoms with WBC count among 9274 (60% females) participants, 35–65 years of age, from a population-based cohort study in north-eastern Iran [53]. Their results suggested an increase in WBC count was linked to increased symptoms severity among men only [53]. Vos et al. examined two markers of systemic inflammation (Neutrophil to Lymphocyte Ratio (NLR) and WBC count) in relation to pharmacotherapy among 87 patients with psychotic depression [54]. Higher NLR was associated with increased response to pharmacotherapy, but not with remission of depression or disappearance of psychotic symptoms, whereas WBC count was not associated with any of these outcomes [54]. A longitudinal study involving >2000 older adult men and women from the Healthy Aging in Neighborhood of Diversity Across the Lifespan Study examined sex-specific relationships of WBC count and the percentage composition of lymphocytes (PL) or neutrophils (PN) with change in depressive symptoms from baseline to follow-up as well as the relationship of depressive symptoms with change in WBC count, PL and PN [75]. In this study, depressive symptoms were assessed using the 20-item CESD scale [75]. Among women, higher WBC count was linked to a faster increase in depressive symptoms, with a slower increase over time in the positive affect subdomain and faster increases in the depressed affect and somatic complaints subdomains [75], whereas baseline score on somatic complaints was positively associated with low PN and high PL, and baseline score on positive affect was inversely related to higher PL [75]. Among men, there was a positive cross-sectional relationship between low WBC count and depressive symptoms, depressed affect, and an inverse cross-sectional relationship with positive affect [75]. However, over time, a low WBC count in men was linked to a higher score on positive affect [75]. Consistent with our study, there was no evidence of a bi-directional relationship between WBC parameters and depressive symptoms [75]. Inconsistencies with the published literature may be explained, in part, by differences in study design, measurements, and population characteristics.

This study has many strengths. First, the WHI database is comprised of a large sample at enrollment with data collection enabling the evaluation of hypothesized relationships while accounting for key confounders. Second, findings from WHI analyses could potentially be generalized to postmenopausal women of diverse racial and ethnic backgrounds living in various geographical areas within the U.S. However, there are several limitations of our study. First, data from WHI-CT and WHI-OS participants were combined in these analyses, although they consist of multiple studies that differ in terms of design and eligibility criteria. For instance, the DM trial consists of two study groups, namely the low-fat diet and control groups. Further analyses suggested that the low-fat diet group who met periodically and attended small group sessions had, on average, similar depressive symptoms scores (0.037 ± 0.036) compared to the control group (*P* = 0.98). By contrast, DM trial participants had, on average, lower depressive symptoms scores as compared to non-DM participants (0.037 ± 0.12 vs. 0.040 ± 0.12, *P* = 0.0024). Second, missing data on exposure, outcome and covariate variables may have resulted in selection bias. Third, measurement errors may have resulted from day-to-day variation in inflammatory markers such as WBC count and the use of a dichotomized depressive symptoms outcome based on the Burnam Algorithm. Fourth, residual confounding due to unmeasured or inadequately measured confounders remains a concern for observational study designs. Fifth, the interpretation of focal estimates for the observed exposure-outcome relationships depends on the way covariates are coded, and these estimates pertain to a prototypical postmenopausal woman with average values for covariates included within group-level or monothetic models. This study which examined postmenopausal women alone pre-supposes that hypothesized relationships may vary according to sex. Further analyses are, therefore, needed to test the robustness of observed relationships within subgroups of postmenopausal women, for the purpose of identifying sample-related contingencies as well as limitations to the generalizability of inferences made. Finally, the WHI is not population-based but involves volunteers at clinical centers, specifically targeting postmenopausal women. Therefore, its generalizability to men as well as younger and less educated women is limited.

In conclusion, a higher WBC count was cross-sectionally associated with a higher prevalence of depressive symptoms and/or use of antidepressants. A faster decline in WBC count over time was observed among women with consistently high as opposed to consistently low levels of depressive symptoms. Distinct cross-sectional and longitudinal findings can be explained by the existence of a positive correlation between depression and inflammatory response at any given timepoint, whereas sustained depression may be associated with immune senescence, as previously suggested by others [51]. Further evaluation of the observed relationships is needed in the context of prospective cohort studies involving older adult men and women, longer follow-up times, with repeated measures on depressive symptoms, antidepressant use and WBC count and its differentials. Comparison of antidepressants to negative control medications on WBC and other inflammatory markers is also warranted.

### Supplementary information

Supplemtal materials

Supplemental tables

## Data Availability

All data will be made available upon request.
