# Supplementary material for: Depressive symptoms and antidepressant use in relation to white blood cell count among postmenopausal women from the Women’s Health Initiative
Source: Transl Psychiatry. 2024 Mar 21;14:157. doi: 10.1038/s41398-024-02872-5 (PMC10958010; doi:10.1038/s41398-024-02872-5)
Supplement: Supplementary file 1 — Supplemtal materials [file 41398_2024_2872_MOESM1_ESM.docx]

**SUPPLEMENTAL MATERIALS**

***Anti-inflammatory medications:*** These are medications with a therapeutic class code that ranges between 661000 and 663099 and are labeled as “Analgesics – Anti-inflammatory”. They include NSAIAs, Cox-2 inhibitors, phenylbutazones, NSAIA combinations, gold compounds, antirheumatic antimetabolites, IL-1 receptor antagonists, anti-TNF-alpha monoclonal antibodies, pyrimidine synthesis inhibitors, soluble tumor necrosis factor inhibitors, miscellaneous antirheumatics, and miscellaneous antirheumatic combinations.

***Dietary Inflammatory Index:*** The DII was developed for the quantitative assessment of diet as a predictor of a wide range of health outcomes including inflammatory cytokines and chronic diseases. The DII which was designed to be universally applied across human studies with adequate dietary assessment, has been used in >200 studies over the past 4 years and forms the basis for 12 meta-analyses^1^. We calculated the DII score based on data from the 122-item Food Frequency Questionnaire (FFQ) which was administered at enrollment to evaluate average dietary intake, including vitamin and mineral supplements, over the past 3 months^2-10^. Results of this WHI FFQ were comparable to four 24-hour dietary recalls as well as 4 days of food diaries within the same population^4, 10^. The University of Minnesota’s Nutrition Data System for Research which is based on the U.S. Department of Agriculture Standard Reference releases and manufacturer information was used to estimate each participant’s consumption of specific nutrients^4, 9, 10^. The original DII score was developed based on a review of 1943 studies that were published through 2010 and examined a total of 45 components (food items or nutrients) in relation to inflammatory markers, which included interleukin (IL)-1β, IL-4, IL-6, IL-10, tumor necrosis factor-α (TNFα), and CRP^4^. Each food item or nutrient was assigned a specific score according to its pro-inflammatory (+1), anti-inflammatory (-1), or null (0) effect while taking number of publications and study design into consideration. For each DII component, a Z-score was calculated by subtracting a global average intake from the actual food item or nutrient intake and dividing by a global standard deviation as described elsewhere^9, 11^. After Z-scores were converted to proportions, they were centered by multiplying by 2 and subtracting by 1^9, 11^. These centered proportions were multiplied by their component-specific inflammatory effect score to obtain component-specific DII scores which were added together to calculate a total DII score for each subject^9, 11^. As described in previously conducted WHI studies, we calculated the total DII score based on 32 out of 45 DII components that were available in the FFQ, with the remaining 13 components not likely to contribute substantially to the original DII score^9, 11^. In this study, the total DII score was defined as a continuous variable.

***Burnam algorithm:*** Depressive symptoms in the past week were assessed with a scale that used 6 items from the 20-item Center for Epidemiological Studies Depression Scale (CES-D) and 2 items from the Diagnostic Interview Schedule (DIS). C*ES-D:* Participants are asked how often they have felt each of these feelings during the past week: ‘You felt depressed’, ‘Your sleep was restless’, ‘You enjoyed life (reversed scoring)’, ‘You had crying spells’, ‘You felt sad’, and ‘You felt that people disliked you’. Each item is scored as 0 (rarely or none of the time [1 day]), 1 (some or a little of the time [1-2 days]), 2 (occasionally or a moderate amount of the time [3-4 days]), or 3 (most or all of the time [5-7 days]). *DIS:* ‘In the past year, have you had 2 weeks or more during which you felt sad, blue, or depressed or lost pleasure in things that you usually cared about or enjoyed? (0, no; 1, yes)’, ‘Have you had 2 years or more in your life when you felt depressed or sad most days, even if you felt okay sometimes? (0, no; 1, yes) If yes, have you felt depressed or sad much of the time in the past year? (0, no; 1, yes)’ ^12-16^. Studies involving the Burnam algorithm conducted in the general population, primary care and mental healthcare settings suggested adequate sensitivity and positive predictive values for detecting depressive disorder, especially for recent disorders and those that met full DSM-III criteria^14^.

**References:**

1. Hebert JR, Shivappa N, Wirth MD, Hussey JR, Hurley TG. Perspective: The Dietary Inflammatory Index (DII)-Lessons Learned, Improvements Made, and Future Directions. *Adv Nutr* 2019; **10**(2)**:** 185-195.

2. Beasley JM, Rillamas-Sun E, Tinker LF, Wylie-Rosett J, Mossavar-Rahmani Y, Datta M *et al.* Dietary Intakes of Women's Health Initiative Long Life Study Participants Falls Short of the Dietary Reference Intakes. *Journal of the Academy of Nutrition and Dietetics* 2020; **120**(9)**:** 1530-1537.

3. Orchard T, Yildiz V, Steck SE, Hebert JR, Ma Y, Cauley JA *et al.* Dietary Inflammatory Index, Bone Mineral Density, and Risk of Fracture in Postmenopausal Women: Results From the Women's Health Initiative. *J Bone Miner Res* 2017; **32**(5)**:** 1136-1146.

4. Tabung FK, Steck SE, Liese AD, Zhang J, Ma Y, Caan B *et al.* Association between dietary inflammatory potential and breast cancer incidence and death: results from the Women's Health Initiative. *Br J Cancer* 2016; **114**(11)**:** 1277-1285.

5. Tabung FK, Steck SE, Liese AD, Zhang J, Ma Y, Johnson KC *et al.* Patterns of change over time and history of the inflammatory potential of diet and risk of breast cancer among postmenopausal women. *Breast Cancer Res Treat* 2016; **159**(1)**:** 139-149.

6. Tabung FK, Steck SE, Ma Y, Liese AD, Zhang J, Caan B *et al.* The association between dietary inflammatory index and risk of colorectal cancer among postmenopausal women: results from the Women's Health Initiative. *Cancer Causes Control* 2015; **26**(3)**:** 399-408.

7. Tabung FK, Steck SE, Zhang J, Ma Y, Liese AD, Agalliu I *et al.* Construct validation of the dietary inflammatory index among postmenopausal women. *Ann Epidemiol* 2015; **25**(6)**:** 398-405.

8. Tabung FK, Steck SE, Zhang J, Ma Y, Liese AD, Tylavsky FA *et al.* Longitudinal changes in the dietary inflammatory index: an assessment of the inflammatory potential of diet over time in postmenopausal women. *Eur J Clin Nutr* 2016; **70**(12)**:** 1374-1380.

9. Zuercher MD, Harvey DJ, Santiago-Torres M, Au LE, Shivappa N, Shadyab AH *et al.* Dietary inflammatory index and cardiovascular disease risk in Hispanic women from the Women's Health Initiative. *Nutr J* 2023; **22**(1)**:** 5.

10. Patterson RE, Kristal AR, Tinker LF, Carter RA, Bolton MP, Agurs-Collins T. Measurement characteristics of the Women's Health Initiative food frequency questionnaire. *Ann Epidemiol* 1999; **9**(3)**:** 178-187.

11. Shivappa N, Steck SE, Hurley TG, Hussey JR, Hebert JR. Designing and developing a literature-derived, population-based dietary inflammatory index. *Public Health Nutr* 2014; **17**(8)**:** 1689-1696.

12. Design of the Women's Health Initiative clinical trial and observational study. The Women's Health Initiative Study Group. *Control Clin Trials* 1998; **19**(1)**:** 61-109.

13. Andresen EM, Malmgren JA, Carter WB, Patrick DL. Screening for depression in well older adults: evaluation of a short form of the CES-D (Center for Epidemiologic Studies Depression Scale). *Am J Prev Med* 1994; **10**(2)**:** 77-84.

14. Burnam MA, Wells KB, Leake B, Landsverk J. Development of a brief screening instrument for detecting depressive disorders. *Med Care* 1988; **26**(8)**:** 775-789.

15. Robins LN, Helzer JE, Croughan J, Ratcliff KS. National Institute of Mental Health Diagnostic Interview Schedule. Its history, characteristics, and validity. *Arch Gen Psychiatry* 1981; **38**(4)**:** 381-389.

16. Wassertheil-Smoller S, Shumaker S, Ockene J, Talavera GA, Greenland P, Cochrane B *et al.* Depression and cardiovascular sequelae in postmenopausal women. The Women's Health Initiative (WHI). *Arch Intern Med* 2004; **164**(3)**:** 289-298.
