# Supplementary material for: Depressive symptoms and antidepressant use in relation to white blood cell count among postmenopausal women from the Women’s Health Initiative
Source: Transl Psychiatry. 2024 Mar 21;14:157. doi: 10.1038/s41398-024-02872-5 (PMC10958010; doi:10.1038/s41398-024-02872-5)
Supplement: Supplementary file 2 — Supplemental tables [file 41398_2024_2872_MOESM2_ESM.docx]

**SUPPLEMENTAL TABLES**

**Table S.1.** Multiple linear regression models for the cross-sectional relationship between depressive symptoms and/or antidepressant use and white blood cell count, adjusted for anti-inflammatory medication use and dietary inflammatory index at enrollment (n=125307)

|  | **WBC count (Kcell/ml)** |
| --- | --- |
|  | **Adjusted *** |
|  | **β (95% CI)** |
| *Model I:* |  |
| **Depressive symptoms (Yes vs. No):** | 0.11 (-0.07, 0.29) |
| *Model II:* |  |
| **Antidepressant use (Yes vs. No):** | 0.11 (-0.11, 0.33) |
| *Model III:* |  |
| **Depressive symptoms and/or antidepressant use (Yes vs. No):** | 0.15 (0.00, 0.31) |
| *Model IV:* |  |
| **Depressive symptoms /Antidepressant use (categorical):** |  |
| No depressive symptoms / No antidepressant use † | Ref. |
| Depressive symptoms / No antidepressant use | 0.17 (-0.03, 0.37) |
| No depressive symptoms / Antidepressant use | 0.22 (-0.04, 0.48) |
| Depressive symptoms / Antidepressant use | -0.10 (-0.51, 0.31) |

*Abbreviations:* β = Slope; CI = Confidence interval; WBC = white blood cells; * Adjusted for *WHI component* (WHI-CT, WHI-OS), *socio-demographic characteristics* (age [in years], race [American Indian/Alaska Native, Asian, Native Hawaiian/Other Pacific Islanders, Black, White, More than one race, Unknown/Not reported], ethnicity [Hispanic, non-Hispanic, Unknown/Not reported], education [less than high school, high school, some college, completed college or higher level], household income [< $20,000, $20,000-$49,999, $50,000-$99,999, ≥$100,000], marital status [Married/Partnered, Single, Divorced, Widowed]), *lifestyle characteristics* (smoking status [Never Smoker, Past Smoker, Current Smoker], alcohol consumption [Non-Drinker, Former Drinker, < 1 drink/week, ≥ 1 drink/week], physical activity [Metabolic equivalent-hours/week]), and *health characteristics*, namely, body mass index (BMI) [< 25, 25-<30, ≥ 30 kg/m^2^], comorbid conditions (cardiovascular disease [Yes, No], hypertension [Yes, No], hyperlipidemia [Yes, No], diabetes [Yes, No]) and self-rated health [Excellent/Very Good/Good, Fair/Poor]), anti-inflammatory medication use (Yes vs. No) and dietary inflammatory index (continuous); † Referent category.

**Table S.2.** Multiple linear regression models for the cross-sectional relationship between depressive symptoms and/or antidepressant use and white blood cell count, whereby the categorical definition of self-rated health was included as a covariate (n=125307)

|  | **WBC count (Kcell/ml)** |
| --- | --- |
|  | **Adjusted *** |
|  | **β (95% CI)** |
| *Model I:* |  |
| **Depressive symptoms (Yes vs. No):** | 0.10 (-0.08, 0.28) |
| *Model II:* |  |
| **Antidepressant use (Yes vs. No):** | 0.10 (-0.12, 0.32) |
| *Model III:* |  |
| **Depressive symptoms and/or antidepressant use (Yes vs. No):** | 0.15 (-0.01, 0.30) |
| *Model IV:* |  |
| **Depressive symptoms /Antidepressant use (categorical):** |  |
| No depressive symptoms / No antidepressant use † | Ref. |
| Depressive symptoms / No antidepressant use | 0.16 (-0.037, 0.36) |
| No depressive symptoms / Antidepressant use | 0.21 (-0.05, 0.47) |
| Depressive symptoms / Antidepressant use | -0.11 (-0.52, 0.29) |

*Abbreviations:* β = Slope; CI = Confidence interval; WBC = white blood cells; * Adjusted for *WHI component* (WHI-CT, WHI-OS), *socio-demographic characteristics* (age [in years], race [American Indian/Alaska Native, Asian, Native Hawaiian/Other Pacific Islanders, Black, White, More than one race, Unknown/Not reported], ethnicity [Hispanic, non-Hispanic, Unknown/Not reported], education [less than high school, high school, some college, completed college or higher level], household income [< $20,000, $20,000-$49,999, $50,000-$99,999, ≥$100,000], marital status [Married/Partnered, Single, Divorced, Widowed]), *lifestyle characteristics* (smoking status [Never Smoker, Past Smoker, Current Smoker], alcohol consumption [Non-Drinker, Former Drinker, < 1 drink/week, ≥ 1 drink/week], physical activity [Metabolic equivalent-hours/week]), and *health characteristics*, namely, body mass index (BMI) [< 25, 25-<30, ≥ 30 kg/m^2^], comorbid conditions (cardiovascular disease [Yes, No], hypertension [Yes, No], hyperlipidemia [Yes, No], diabetes [Yes, No]) and self-rated health [Excellent, Very Good, Good, Fair, Poor]); † Referent category.

**Table S.3.** Linear regression model for the cross-sectional relationship between types of antidepressants and white blood cell count at enrollment (n=125307)

|  | **N** | | **WBC count (Kcell/ml)** | |
| --- | --- | --- | --- | --- |
|  |  | | **Unadjusted** | **Adjusted *** |
|  | **Yes** | **No** | **β (95% CI)** | **β (95% CI)** |
| ***α-2 receptor antagonists (Yes vs. No):*** | 20 | 125287 | 0.36 (-4.01, 4.74) | 0.04 (-4.32, 4.44) |
| ***Monoamine oxidase inhibitors (Yes vs. No):*** | 35 | 125272 | -0.21 (-3.52, 3.10) | -0.50 (-3.81, 2.79) |
| ***Modified cyclics (Yes vs. No):*** | 695 | 124612 | -0.055 (-0.79, 0.69) | -0.22 (-0.96, 0.52) |
| ***Selective serotonin reuptake inhibitors (Yes vs. No):*** | 4306 | 121001 | 0.45 (0.15, 0.75) | 0.21 (-0.09, 0.51) |
| ***Serotonin-norepinephrine reuptake inhibitors***  ***(Yes vs. No):*** | 0 | 125307 | NA | NA |
| ***Tricyclic agents (Yes vs. No):*** | 3341 | 121966 | 0.26 (-0.08, 0.60) | 0.04 (-0.31, 0.38) |
| ***Miscellaneous antidepressants (Yes vs. No):*** | 658 | 124649 | 0.01 (-0.75, 0.78) | -0.25 (-1.02, 0.51) |
| ***Antidepressant combinations (Yes vs. No):*** | 0 | 125307 | NA | NA |

*Abbreviations:* β = Slope; CI = Confidence Interval; NA = No data available; WBC = white blood cells; * Adjusted for *WHI component* (WHI-CT, WHI-OS), *socio-demographic characteristics* (age [in years], race [American Indian/Alaska Native, Asian, Native Hawaiian/Other Pacific Islanders, Black, White, More than one race, Unknown/Not reported], ethnicity [Hispanic, non-Hispanic, Unknown/Not reported], education [less than high school, high school, some college, completed college or higher level], household income [< $20,000, $20,000-$49,999, $50,000-$99,999, ≥$100,000], marital status [Married/Partnered, Single, Divorced, Widowed]), *lifestyle characteristics* (smoking status [Never Smoker, Past Smoker, Current Smoker], alcohol consumption [Non-Drinker, Former Drinker, < 1 drink/week, ≥ 1 drink/week], physical activity [Metabolic equivalent-hours/week]), and *health characteristics*, namely, body mass index (BMI) [< 25, 25-<30, ≥ 30 kg/m^2^], comorbid conditions (cardiovascular disease [Yes, No], hypertension [Yes, No], hyperlipidemia [Yes, No], diabetes [Yes, No]) and self-rated health [Excellent/Very Good/Good, Fair/Poor]).

**Table S.4.** Logistic regression models for the cross-sectional relationship of white blood cell count tertiles as predictors of depressive symptoms and/or antidepressant use at enrollment (n=125307) *

|  | **N** | | **Unadjusted** | **Adjusted **** |
| --- | --- | --- | --- | --- |
|  |  | | **OR (95% CI)** | **OR (95% CI)** |
| **Model I – Depressive symptoms (Yes vs. No):** | **Yes** | **No** |  |  |
| *WBC count (Kcell/ml):* |  |  |  |  |
| 1^st^ tertile | 3779 | 36549 | Ref. | Ref. |
| 2^nd^ tertile | 4447 | 38693 | 1.11 (1.06, 1.16) | 1.04 (0.99, 1.09) |
| 3^rd^ tertile | 5132 | 36707 | 1.35 (1.29, 1.41) | 1.07 (1.02, 1.13) |
| **Model II – Antidepressant use (Yes vs. No):** | **Yes** | **No** |  |  |
| *WBC count (Kcell/ml):* |  |  |  |  |
| 1^st^ tertile | 2346 | 37982 | Ref. | Ref. |
| 2^nd^ tertile | 2688 | 40452 | 1.08 (1.02, 1.14) | 0.95 (0.90, 1.02) |
| 3^rd^ tertile | 3328 | 38511 | 1.39 (1.32, 1.47) | 1.04 (0.98, 1.11) |
| **Model III – Depressive symptoms and/or antidepressant use (Yes vs. No):** | **Yes** | **No** |  |  |
| *WBC count (Kcell/ml):* |  |  |  |  |
| 1^st^ tertile | 5502 | 34826 | Ref. | Ref. |
| 2^nd^ tertile | 6382 | 36758 | 1.09 (1.06, 1.14) | 1.01 (0.97, 1.05) |
| 3^rd^ tertile | 7470 | 34369 | 1.37 (1.32, 1.43) | 1.07 (1.03, 1.12) |

*Abbreviations:* CI = Confidence Interval; OR = Odds Ratio; WBC = white blood cells; * WBC count were defined in tertiles; ** Adjusted for *WHI component* (WHI-CT, WHI-OS), *socio-demographic characteristics* (age [in years], race [American Indian/Alaska Native, Asian, Native Hawaiian/Other Pacific Islanders, Black, White, More than one race, Unknown/Not reported], ethnicity [Hispanic, non-Hispanic, Unknown/Not reported], education [less than high school, high school, some college, completed college or higher level], household income [< $20,000, $20,000-$49,999, $50,000-$99,999, ≥$100,000], marital status [Married/Partnered, Single, Divorced, Widowed]), *lifestyle characteristics* (smoking status [Never Smoker, Past Smoker, Current Smoker], alcohol consumption [Non-Drinker, Former Drinker, < 1 drink/week, ≥ 1 drink/week], physical activity [Metabolic equivalent-hours/week]), and *health characteristics*, namely, body mass index (BMI) [< 25, 25-<30, ≥ 30 kg/m^2^], comorbid conditions (cardiovascular disease [Yes, No], hypertension [Yes, No], hyperlipidemia [Yes, No], diabetes [Yes, No]) and self-rated health [Excellent/Very Good/Good, Fair/Poor]).

**Table S.5.** Logistic regression models for the cross-sectional relationship of white blood cell count quintiles as predictors of depressive symptoms and/or antidepressant use at enrollment (n=125307) *

|  | **N** | | **Unadjusted** | **Adjusted **** |
| --- | --- | --- | --- | --- |
|  |  | | **OR (95% CI)** | **OR (95% CI)** |
| **Model I – Depressive symptoms (Yes vs. No):** | **Yes** | **No** |  |  |
| *WBC count (Kcell/ml):* |  |  |  |  |
| 1^st^ quintile | 2203 | 21694 | Ref. | Ref. |
| 2^nd^ quintile | 2640 | 24456 | 1.06 (1.00, 1.13) | 1.04 (0.98, 1.10) |
| 3^rd^ quintile | 2438 | 21404 | 1.12 (1.06, 1.19) | 1.04 (0.98, 1.11) |
| 4^th^ quintile | 2806 | 22198 | 1.24 (1.17, 1.32) | 1.08 (1.02, 1.16) |
| 5^th^ quintile | 3271 | 22197 | 1.45 (1.37, 1.53) | 1.08 (1.02, 1.15) |
| **Model II – Antidepressant use (Yes vs. No):** | **Yes** | **No** |  |  |
| *WBC count (Kcell/ml):* |  |  |  |  |
| 1^st^ quintile | 1362 | 22535 | Ref. | Ref. |
| 2^nd^ quintile | 1602 | 25494 | 1.04 (0.96, 1.12) | 0.95 (0.88, 1.03) |
| 3^rd^ quintile | 1478 | 22364 | 1.09 (1.01, 1.18) | 0.94 (0.87, 1.02) |
| 4^th^ quintile | 1737 | 23267 | 1.23 (1.15, 1.33) | 0.98 (0.92, 1.07) |
| 5^th^ quintile | 2183 | 23285 | 1.55 (1.44, 1.66) | 1.07 (0.99, 1.16) |
| **Model III – Depressive symptoms and/or antidepressant use (Yes vs. No):** | **Yes** | **No** |  |  |
| *WBC count (Kcell/ml):* |  |  |  |  |
| 1^st^ quintile | 3180 | 20717 | Ref. | Ref. |
| 2^nd^ quintile | 3839 | 23257 | 1.08 (1.02, 1.13) | 1.03 (0.98, 1.09) |
| 3^rd^ quintile | 3506 | 20336 | 1.12 (1.07, 1.18) | 1.02 (0.97, 1.07) |
| 4^th^ quintile | 4044 | 20960 | 1.25 (1.19, 1.32) | 1.06 (1.01, 1.12) |
| 5^th^ quintile | 4785 | 20683 | 1.51 (1.44, 1.58) | 1.09 (1.04, 1.16) |

*Abbreviations:* CI = Confidence Interval; OR = Odds Ratio; WBC = white blood cells; * WBC count were defined in quintiles; ** Adjusted for *WHI component* (WHI-CT, WHI-OS), *socio-demographic characteristics* (age [in years], race [American Indian/Alaska Native, Asian, Native Hawaiian/Other Pacific Islanders, Black, White, More than one race, Unknown/Not reported], ethnicity [Hispanic, non-Hispanic, Unknown/Not reported], education [less than high school, high school, some college, completed college or higher level], household income [< $20,000, $20,000-$49,999, $50,000-$99,999, ≥$100,000], marital status [Married/Partnered, Single, Divorced, Widowed]), *lifestyle characteristics* (smoking status [Never Smoker, Past Smoker, Current Smoker], alcohol consumption [Non-Drinker, Former Drinker, < 1 drink/week, ≥ 1 drink/week], physical activity [Metabolic equivalent-hours/week]), and *health characteristics*, namely, body mass index (BMI) [< 25, 25-<30, ≥ 30 kg/m^2^], comorbid conditions (cardiovascular disease [Yes, No], hypertension [Yes, No], hyperlipidemia [Yes, No], diabetes [Yes, No]) and self-rated health [Excellent/Very Good/Good, Fair/Poor]).

**Table S.6.** Logistic regression models for the cross-sectional relationship of white blood cell count with linear, quadratic and cubic terms as predictors of depressive symptoms and/or antidepressant use at enrollment (n=125307) *

|  | **Unadjusted** | **Adjusted **** |
| --- | --- | --- |
|  | **OR (95% CI)** | **OR (95% CI)** |
| **Model I – Depressive symptoms (Yes vs. No):** |  |  |
| *WBC count (Kcell/ml):* |  |  |
| Linear | 0.95 (0.94, 0.96) | 0.99 (0.98, 1.00) |
| Quadratic | 1.00 (1.00, 1.00) | 1.00 (1.00, 1.00) |
| Cubic | 1.00 (1.00, 1.00) | 1.00 (1.00, 1.00) |
| **Model II – Antidepressant use (Yes vs. No):** |  |  |
| *WBC count (Kcell/ml):* |  |  |
| Linear | 0.95 (0.94, 0.96) | 0.99 (0.98, 1.00) |
| Quadratic | 1.00 (1.00, 1.00) | 1.00 (1.00, 1.00) |
| Cubic | 1.00 (1.00, 1.00) | 1.00 (1.00, 1.00) |
| **Model III – Depressive symptoms and/or antidepressant use (Yes vs. No):** |  |  |
| *WBC count (Kcell/ml):* |  |  |
| Linear | 0.94 (0.93, 0.95) | 0.99 (0.98, 1.00) |
| Quadratic | 1.00 (1.00, 1.00) | 1.00 (1.00, 1.00) |
| Cubic | 1.00 (1.00, 1.00) | 1.00 (1.00, 1.00) |

*Abbreviations:* CI = Confidence Interval; OR = Odds Ratio; WBC = white blood cells; * WBC count is defined as continuous variable, with linear, quadratic, and cubic terms, and all these terms are centered around the mean value of WBC count (6.11 Kcell/ml); ** Adjusted for *WHI component* (WHI-CT, WHI-OS), *socio-demographic characteristics* (age [in years], race [American Indian/Alaska Native, Asian, Native Hawaiian/Other Pacific Islanders, Black, White, More than one race, Unknown/Not reported], ethnicity [Hispanic, non-Hispanic, Unknown/Not reported], education [less than high school, high school, some college, completed college or higher level], household income [< $20,000, $20,000-$49,999, $50,000-$99,999, ≥$100,000], marital status [Married/Partnered, Single, Divorced, Widowed]), *lifestyle characteristics* (smoking status [Never Smoker, Past Smoker, Current Smoker], alcohol consumption [Non-Drinker, Former Drinker, < 1 drink/week, ≥ 1 drink/week], physical activity [Metabolic equivalent-hours/week]), and *health characteristics*, namely, body mass index (BMI) [< 25, 25-<30, ≥ 30 kg/m^2^], comorbid conditions (cardiovascular disease [Yes, No], hypertension [Yes, No], hyperlipidemia [Yes, No], diabetes [Yes, No]) and self-rated health [Excellent/Very Good/Good, Fair/Poor]).

**Table S.7.** Linear regression models for change in WBC count between enrollment and 3-year follow-up visits in relation to patterns of depressive symptoms and antidepressant use between enrollment and 3-year follow-up visits, whereby adjusted models were controlled for age, body mass index, and self-rated health

|  | **Change in WBC count (Kcell/ml)** | | |
| --- | --- | --- | --- |
|  | **β (95% CI)** | | |
|  | **N** | **Unadjusted *** | **Adjusted *** |
| **Depressive symptoms (n=56411):** |  |  |  |
| Consistently had no depressive symptoms | 47774 | Ref. | Ref. |
| Depressive symptoms at enrollment only | 3453 | 0.16 (-0.30, 0.62) | 0.16 (-0.29, 0.63) |
| Depressive symptoms at follow-up only | 3069 | -0.15 (-0.63, 0.33) | -0.14 (-0.62, 0.35) |
| Consistently had depressive symptoms | 2115 | -0.74 (-1.32, -0.16) | -0.74 (-1.33, -0.14) |
| **Antidepressant use (n=58024):** |  |  |  |
| Consistent non-user (enrollment and follow-up) | 52168 | Ref. | Ref. |
| Enrollment user only | 2596 | -0.26 (-0.79, 0.26) | -0.24 (-0.77, 0.28) |
| Follow-up user only | 1344 | 0.13 (-0.59, 0.86) | 0.15 (-0.57, 0.88) |
| Consistent user (enrollment and follow-up) | 1916 | 0.12 (-0.48, 0.74) | 0.16 (-0.45, 0.78) |

*Abbreviations:* β = Slope; CI = confidence intervals; WBC = White blood cells; ** Adjusted for age [in years], body mass index (BMI) [[< 25, 25-<30, ≥ 30 kg/m^2^], and self-rated health [Excellent/Very Good/Good, Fair/Poor]).

**Table S.8.** Mixed-effects linear regression models for the relationship between WBC count (enrollment and change) and depressive symptoms score at enrollment (n=57690)

|  | **WBC count (Kcell/ml)** | |
| --- | --- | --- |
|  | **β (95% CI)** | |
|  | **Unadjusted** | **Adjusted *** |
| Depressive symptoms score at enrollment | 0.76 (0.08, 1.44) | 0.14 (0.54, 0.83) |
| Depressive symptoms score at enrollment x Visit | -0.042 (-0.40, 0.3) | 0.19 (-0.34, 0.39) |

*Abbreviations:* β = Slope; CI = confidence intervals; WBC = White blood cells; * Adjusted for *WHI component* (WHI-CT, WHI-OS), *socio-demographic characteristics* (age [in years], race [American Indian/Alaska Native, Asian, Native Hawaiian/Other Pacific Islanders, Black, White, More than one race, Unknown/Not reported], ethnicity [Hispanic, non-Hispanic, Unknown/Not reported], education [less than high school, high school, some college, completed college or higher level], household income [< $20,000, $20,000-$49,999, $50,000-$99,999, ≥$100,000], marital status [Married/Partnered, Single, Divorced, Widowed]), *lifestyle characteristics* (smoking status [Never Smoker, Past Smoker, Current Smoker], alcohol consumption [Non-Drinker, Former Drinker, < 1 drink/week, ≥ 1 drink/week], physical activity [Metabolic equivalent-hours/week]), and *health characteristics*, namely, body mass index (BMI) [< 25, 25-<30, ≥ 30 kg/m^2^], comorbid conditions (cardiovascular disease [Yes, No], hypertension [Yes, No], hyperlipidemia [Yes, No], diabetes [Yes, No]) and self-rated health [Excellent/Very Good/Good, Fair/Poor]).

**Table S.9.** Mixed-effects linear regression models for the relationship between depressive symptoms score (enrollment and change) and WBC count at enrollment (n=57690)

|  | **Depressive symptoms score** | |
| --- | --- | --- |
|  | **β (95% CI)** | |
|  | **Unadjusted** | **Adjusted *** |
| WBC count at enrollment | 0.00020  (0.00010, 0.00030) | 0.000076  (-0.00002, 0.000177) |
| WBC count at enrollment x Visit | -0.00005  (-0.00010, 0.000006) | 0.000025  (-0.00007, 0.000029) |

*Abbreviations:* β = Slope; CI = confidence intervals; WBC = White blood cells; * Adjusted for *WHI component* (WHI-CT, WHI-OS), *socio-demographic characteristics* (age [in years], race [American Indian/Alaska Native, Asian, Native Hawaiian/Other Pacific Islanders, Black, White, More than one race, Unknown/Not reported], ethnicity [Hispanic, non-Hispanic, Unknown/Not reported], education [less than high school, high school, some college, completed college or higher level], household income [< $20,000, $20,000-$49,999, $50,000-$99,999, ≥$100,000], marital status [Married/Partnered, Single, Divorced, Widowed]), *lifestyle characteristics* (smoking status [Never Smoker, Past Smoker, Current Smoker], alcohol consumption [Non-Drinker, Former Drinker, < 1 drink/week, ≥ 1 drink/week], physical activity [Metabolic equivalent-hours/week]), and *health characteristics*, namely, body mass index (BMI) [< 25, 25-<30, ≥ 30 kg/m^2^], comorbid conditions (cardiovascular disease [Yes, No], hypertension [Yes, No], hyperlipidemia [Yes, No], diabetes [Yes, No]) and self-rated health [Excellent/Very Good/Good, Fair/Poor]).
